# Assessing the Effectiveness of Functional Genetic Screens for the Identification of Bioactive Metabolites 

**DOI:** 10.3390/md11010040

**Published:** 2012-12-27

**Authors:** Anahit Penesyan, Francesco Ballestriero, Malak Daim, Staffan Kjelleberg, Torsten Thomas, Suhelen Egan

**Affiliations:** 1 School of Biotechnology and Biomolecular Sciences and Centre for Marine Bio-Innovation, University of New South Wales, Sydney 2052, New South Wales, Australia; E-Mails: anahit.penesyan@mq.edu.au (A.P.); f.ballestriero@unsw.edu.au (F.B.); malak.daim@gmail.com (M.D.); s.kjelleberg@unsw.edu.au (S.K.); t.thomas@unsw.edu.au (T.T.); 2 Department of Chemistry and Biomolecular Sciences, Macquarie University, Sydney 2109, New South Wales, Australia; 3 The Singapore Center on Life Sciences Engineering, Nanyang Technological University, Singapore, Singapore

**Keywords:** antibacterial, antinematode, functional genomic screen, fosmid libraries, marine bacteria, marine bioactives, *Caenorhabditis elegans*

## Abstract

A common limitation for the identification of novel activities from functional (meta) genomic screens is the low number of active clones detected relative to the number of clones screened. Here we demonstrate that constructing libraries with strains known to produce bioactives can greatly enhance the screening efficiency, by increasing the “hit-rate” and unmasking multiple activities from the same bacterial source.

## 1. Introduction

Functional metagenomics, which includes the cloning of total DNA obtained from an environment into the host bacterium and screening the recombinant clones for a desired activity, is currently a widely used tool for the discovery of novel enzyme and bioactive metabolites [1]. Some of the successes of these functional screens are illustrated by the discovery of antibiotics, such as terragine A [2], bioactive *N*-acyl-tyrosine derivatives [3] as well as indirubin [4]. 

Functional screens have also assisted in the understanding of the genomic bases of biosynthetic pathways underlying the production of bioactive compounds in single organisms. As an example, Burke *et al.* (2007) [5] screened an *Escherichia coli* fosmid library constructed with genomic DNA from the marine bacterium *P. tunicata*, which is known for its ability to produce various bioactive compounds [6]. Clones producing the antifungal compound tambjamine were identified and a biosynthetic pathway was proposed based on the expressed genes required for tambjamine production [5].

Success of such screens is obviously dependent on the ability of the host organisms to express and produce the desired activity. This will be limited by such factors as transcription initiation, codon usage and protein folding, which are well-studied issues for heterologous protein expression in *E. coli* and other hosts [7,8,9]. In addition, heterologous expression of certain genes can be toxic to the host [10] and this is particularly relevant for screens that search for antibiotic activities. Finally, for metagenomic screens, one also has to consider that the desired activities (such as the production of antibiotics) are not evenly distributed among all members of the community sampled, but might reside in rare organisms [8]. Discovery of such “rare” activities would thus require the screening of a large number of clones. All these factors are likely to conspire to cause the low discovery rates (“hit rates”) typically observed for metagenomic screens [11,12], which is rarely exceeding one positive in 10,000 screened clones (0.01%) [3,13] and often being lower (e.g., 0.00013%) [14]. However, it is not clear which of these aspects mentioned above are the major limiting factors.

In this study, we addressed this issue by investigating the screening efficiency for libraries that are enriched for bioactive-producing genomes. By comparing the hit rate to other metagenomic screens we aim to identify if the expression of bioactives *per se* or the abundance of genes encoding for such activities is the major limiting factor for the success of functional genetic screens.

## 2. Results and Discussion

**Figure 1:**
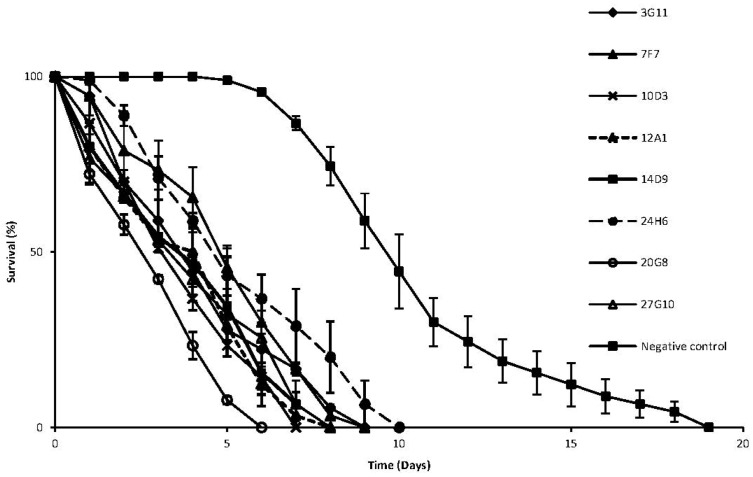
Killing kinetics of the eight antinematode clones. A randomly chosen clone from the library with no activity was used as a negative control. Bars represent standard deviation of three replicates.

To assess the efficiency of our functional screens we constructed a fosmid library from the DNA of six marine bacterial isolates known to have antibacterial properties [15], expressed the library in *E. coli* and screened for activity against both bacteria and the nematode *Caenorhabditis elegans*. Our screens identified both antibacterial and antinematode clones (Table 1). Antibacterial activity was observed in eight clones. The selective grazing assay with *C. elegans* also resulted in eight positive clones, with five of them also possessing antibacterial activity. Clones with antinematode activity were further characterized in the nematode killing assay [16] which revealed a severe killing phenotype (LT_50_ < 5 days) for all the eight positives, with clone 20G8 being the most active in shortening the worms’ life span from 19 (non-toxic *E. coli* clone) to 6 days (Figure 1). The detection of both antibacterial and antinematode activities in five clones suggests that the compounds or enzymes encoded by the fosmid clones could possess a broad range of activity against bacteria and nematodes. Alternatively, two separate compounds or enzymes could be encoded on the same 35 kb fosmid. 

**Table 1:** Bioactive fosmid clones and their original producer strain.

Library clone number	GenBank no.	Fosmid size (bp)	Antibacterial activity	Antinematode activity	Parental strain *
3G11	JX523949	24 296	+	+	D250
7F7	KC211770	34 039	−	+	U95
10D3	JX523951	36 314	+	+	D323
12A1	JX523952	32 547	+	+	D250
14D9	JX523953	19 858	+	+	D323
24H6	KC211769	15 952	−	+	D323
27G10	KC211768	33 970	−	+	U95
20G8	JX523957	25 000	+	+	D250
15E10	JX523954	30 000	+	+	D250
23H6	JX523958	23 000	+	+	D250
19F10	JX523956	29 696	+	−	U95
16B12	JX523955	39 325	+	−	D323
9E12	JX523950	37 944	+	−	D323

* see Table 2 for strain details.

Of the 13 active (*i.e.*, antibacterial and/or antinematode) clones identified, three (15E10, 20G8, 23H6) shared overlapping nucleotide sequences, while the remaining 10 clones were unique in terms of their sequences (see Table 1 for GenBank accession numbers). Thus for each screen (*i.e.*, antibacterial and antinematode) this equates to 11 active clones with unique genomic regions for the 2880 clones screened (~0.4%), which is considerably higher than previous metagenomic screens (see above). For example, a hit-rate of 0.001% was recently achieved for a screen of the metagenome created from the microbial community of *U. australis* [17], from which some of the isolates used in this study were derived. Thus our results would suggest that a pre-selection of bioactive-producing genomes helps with improving hit-rates and that the low abundance of organisms that encode such activities could be a limitation to the success of metagenomic screens. 

In addition, our data suggests that this method is able to detect genes and gene clusters for both known bioactive compounds, as well as detect genes encoding for the production of potentially novel bioactivities. For example, overlapping fosmids 15E10, 20G8, 23H6 in addition to encoding for both antibacterial and antinematode activities resulted in the production of a purple pigment when expressed in *E. coli*. Genetic analysis of each of these fosmids identified a cluster of five genes (*vioA–vioE*) previously characterized as the biosynthetic pathway for the purple pigment and known antibiotic violacein produced by other bacteria such as *Chromobacterium violaceum* and *P. tunicata* [18,19]. As another example, sequencing of fosmid 19F10, originating from the bacterial isolate U95—the type strain for the newly described genus and species *Epibacterium ulvae* [20], identified a gene with sequence similarity to a non-ribosomal peptide synthetase (NRPS) gene with homology to the NRPS gene *bpsA* from *Streptomyces lavendulae* [21] and *indC* from *Erwinia chrysanthemi* [22]. Both BpsA and IndC are annotated as indigoidine synthase, which is responsible in part for the production of the blue pigment indigoidine. Notably, the other genes required for the biosynthesis of indigoidine were absent from the 19F10 fosmid and expression of this fosmid did not result in the production of a blue pigment indicating that the NRPS of 19F10 may be responsible for expression of something other than indigoidine. There is strong evidence in the literature highlighting the role of NRPS in the production of various secondary metabolites with biological activities ranging from antibiotics and toxins to iron scavenging siderophores (as reviewed in [23]). Therefore, this gene is a primary candidate responsible for the production of a potentially novel antibacterial compound produced by a newly characterized bacterium. In addition to a NRPS, the 19F10 fosmid also encodes the genes for various transporters, such as the ATP-binding cassette (ABC) transporters, a major facilitator superfamily (MFS) permease, as well as genes encoding proteins for the type VI secretion system; these may potentially be involved in the secretion of bioactive compound. Moreover many of the genes detected on the active fosmids encoded for hypothetical proteins with little homology to previously characterized sequences, once again highlighting the opportunity to uncover new biologically active metabolites. Future studies will aim to elucidate further details of the chemical or biological nature for the activities found, however the unique gene sequences for the majority of clones identified in this study supports the hypothesis that screening efficiency can be greatly improved by the use of expression libraries that are enriched for bioactive-producing genomes.

For both the antibacterial and antinematode activity the clones were traced back to only three (50%) of the bacterial strains (D250, D323 and U95) used to construct the library. As mentioned above, this might be due to difficulties with the expression of foreign genes, particularly from distantly related organisms, in *E. coli*. Indeed a recent assessment of functional gene expression from soil metagenomes discovered several bioactive clones, which were only expressed in *Streptomyces lividans* (phylum Actinobacteria), but not in *E. coli* (phylum Proteobacteria) [24]. The limited expression of genes from strains distantly related to *E. coli* is further supported by our data as for the three phyla represented in our library (Actinobacteria, Bacteriodetes and Proteobacteria) (see Table 2), active *E. coli* clones were only detected for source strains belonging to the Proteobacteria.

**Table 2:** Bacterial strains used in the construction of the fosmid library in this study.

Strain ID	GenBank no.	Isolation source	Closest relative	Phylum	% Identity
U95	FJ440958	*Ulva australis*	Uncultured alpha-proteobacterium, JN874385	Proteobacteria	98
U140	FJ440963	*Ulva australis*	*Micrococcus luteus*, JQ795852	Actinobacteria	99
U156	FJ440965	*Ulva australis*	Gamma-proteobacterium D261, FJ440978	Proteobacteria	99
D250	FJ440973	*Delisea pulchra*	Gamma-proteobacterium D259, FJ440977	Proteobacteria	99
D295	FJ440982	*Delisea pulchra*	*Flavobacteriaceae* bacterium SW058, AF493683	Bacteroidetes	98
D323	FJ440988	*Delisea pulchra*	*Pseudovibrio* sp. Pv348, 1413, HE818384	Proteobacteria	100

Our screens also detected multiple antibacterial and/or antinematode activities from the same source organism. For example, the genetic screened revealed five genetically distinct antibacterial fosmids for strain D323, which would suggest that five different antibacterial activities are encoded in the genome of isolate D323. Thus a functional genetic screen could help to “tease apart” multiple activities within a source organism and reveal previously unknown activities, something that is difficult to do with classical approaches, such as knock-out genetics. A functional screening approach is thus useful for the exploration of “metabolically talented” strains [25,26] able to produce a wide range of secondary metabolites and may further assist in the separation and identification of compounds by using the host strain without the expressed fosmid as a reference during chemical analysis. 

## 3. Experimental Section

Six marine bacterial isolates known to have antibacterial activity were used to construct a combined fosmid library and screened for antibacterial and antinematode activities. Specifically, genomic DNA was extracted according to the XS DNA extraction protocol [27] from bacterial strains, which were previously isolated from the surface of the marine algae *Ulva australis* and *Delisea pulchra* and which comprised of both phenotypically and phylogenetically distinct groups [15] (Table 2). DNA was pooled in equimolar amounts, randomly sheared, size selected by gel purification (~35 kb) and cloned into the fosmid pCCFOS1 (Epicentre Biotechnologies) according to the manufacturers’ instructions. Fosmid clones were stored and maintained at a single copy number, but induced to high copy number (10–50 per cell) through the addition of L-arabinose (0.02%) to the growth medium during screens.

Clones were screened in an overlay assay using *Staphylococcus aureus* and *Neisseria canis* as target strains [15] as well as in a selective grazing assay and subsequent toxicity assay using the nematode *C. elegans* [16]. The 2880 clones (average insert size ~35 kb) were screened, which covered approximately 100 Mb of genomic DNA. In line with previous studies [5,17,18,24] and assuming an average genome size of 3.5 Mbp [28,29], this number of clones would cover all six genomes on average 4.5 fold. Screens were repeated three times after which thirteen clones were selected which consistently had high levels of either antibacterial or antinematode activities. Fosmids were extracted from these clones, shotgun sequenced (Craig Venter Institute, Rockville, MD, USA) and then annotated (supplementary material). Fosmids were linked back to the original bacterial strain via PCR (supplementary material). 

## 4. Conclusions

Heterologous expression and possible toxic effects on the host remain clear limitations for the identification of bioactivities in genetic screens [9]. However the relatively high hit rate observed in our study indicates that the scarcity of DNA encoding for bioactivities might be a significant limitation for metagenomic screens. Whilst the higher hit rate using a pre-selection of active strains is not necessarily surprising, to our knowledge, this is the first study to experimentally address the abundance of bioactive genes as a limitation to functional metagenomic screens. Studies have shown that metagenomic libraries constructed of DNA pooled from cultured isolates is effective in detecting antibiotic resistance phenotypes [30] and more recently pigment production and hemolytic activity [24]. However, neither of these studies used cultured isolates known to have these respective activities. Our results further highlight the need for a targeted application of functional metagenomics to environments in which, for example, ecological factors select for high abundance of bioactive-producing organisms. 

## Supplementary Material

### Analysis of Fosmids

Sequencing reads obtained from ABI3730XL and the Roche Titanium FLX DNA sequencer, were trimmed for vector contamination (*i.e.*, pCC1FOS) and low quality, using the Phred/Phrap/Consed software pipeline [31]. Reads from the shotgun library were assembled with Phrap and the assembly manually checked in Consed. Gaps and low quality regions were closed by targeted PCRs and sequencing. Overlapping regions between the fosmids were identified from the final curated assemblies and from pairwise BLAST searches. Open reading frames (ORFs) were identified with the program MetaGene [32,33]. All predicted ORFs were searched (using an in-house pipeline) [34] against the Swiss-Prot database [35], the Institute of Genome Research Family (TIGRFAM) database [36], the Kyoto Encyclopaedia of Genes and Genomes (KEGG) database [37], and the Cluster of Orthologous Group of proteins (COG) database [38] to obtain a functional annotation.

### Identification of Fosmid Parental Strains

PCR amplification was used to identify which parent genome the selected fosmids belonged to. Briefly, specific primer pairs were designed based on each fosmid sequence (Table S1). Genomic DNA of each of the six isolates used in the fosmid library construction (U95, U140, U156, D250, D295, D323) was used as template for amplification using the following conditions. Amplification was performed in 20 μL reaction mixes each containing 50 ng of genomic DNA; 2 μL REDtaq buffer (Sigma-Aldrich, St. Louis, MO, USA), 2.5 mM each dNTP (Roche, Penzberg, Germany), 12.5 pmol of each of the forward and reverse primers (Table S1) (Sigma-Aldrich, St. Louis, MO, USA), 0.5 μL of 10% BSA (w/v, New England Biolabs, Ipswich, MA, USA) and molecular grade water (Eppendorf, Hamburg, Germany). One unit of REDtaq polymerase (Sigma-Aldrich, St. Louis, MO, USA) was added at the Hot Start, after the initial thermal ramp. The PCR conditions were 94 °C for 3 min, then 25 cycles each of 1 min at 94 °C, 1 min at 55 °C, and 2 min at 72 °C. A final extension step of 72 °C for 6 min was performed. Amplified DNA fragments were subjected to agarose gel electrophoresis to check for the presence of amplification products (data not shown).

**Table S1:** Primer pairs used for fosmid parent strain identification and their expected product lengths.

Fosmid	Primer pairs	Sequence (5′ to 3′)	Product length
3G11	3G11 forward	GGC TAG AGG CGT TGC GTA TTG TGC	679 bp
	3G11 reverse	CTT TAA AGG CGC CGG GCT CCA TCT	
7F7	7F7 forward	AAC CTG CCA GAT ACC AAA CG	1728 bp
	7F7 reverse	GGT CAA CCG GAA CAC AGA GT	
9E12	9E12 forward	TGC TGA AGC GGA AGT GGA GTA TGA	388 bp
	9E12 reverse	CGG CAC GTT GAA GTC GAA GTA GTC	
10D3	10D3 forwards	CTA TGA TCA CGA CCA GCA CAC GAG	571 bp
	10D3 reverse	ACC AGG TCC GAG CCA TCT ACA CAA	
12A1	12A1 forward	ACA GCG GTG GTC ATT ATT GGA ACG	432 bp
	12A1 reverse	GGC GGT GTG AAA GCG GTG ATA GTC	
14D9	14D9 forward	GGC ACA CGG CTC TTC ATC TTC ACA	532 bp
	14D9 reverse	GCC GCG TTC GTT CCC GTC AC	
24H6	24H6 forward	CGT GAA TGT GGA AGG TGT TG	2228 bp
	24H6 reverse	AAA GAA AGC TTG GCG TTG AA	
15E10	15E10 forward	GCT AAA CTG CCT GAC TTC TAC ACG	509 bp
20G8	15E10 reverse	CTG GAT ACT GCT GGT TTG ACT ACG	
23H6			
16B12	16B12 forward	CTC TTT ACG CCC AGT GAT TCC	613 bp
	16B12 reverse	TTA TTT GCG TGT TCC TCG TCT ATT	
19F10	19F10 forward	ACA TCA TCG CCG CTA AGG TA	772 bp
	19F10 reverse	TAT GGG ATT CTG TTG TTT CGT AA	
27G10	27G10 forward	AGC GGC TTA CCT CAA GAA CA	1803 bp
	27G10 reverse	GCT GAG AAC CCA GAA AGT CG	
